# Supplementary material for: Expression-Based Diagnosis, Treatment Selection, and Drug Development for Breast Cancer
Source: Int J Mol Sci. 2023 Jun 23;24(13):10561. doi: 10.3390/ijms241310561 (PMC10342177; doi:10.3390/ijms241310561)
Supplement: Supplementary file 1 [file ijms-24-10561-s001.zip › Supplementary File S1.pdf]

Table S1. Genes and primers on microfluidic low-density arrays in RT-PCR assays of the gene signature.

Gene	Primer
TOMM70A	Hs00207896_m1
MCF2	Hs00180467_m1
RAD52 homolog	Hs00172536_m1
MCM2	Hs00170472_m1
C18B11	Hs00398863_m1
SEC13L	Hs00229687_m1
SLC25A5	Hs00854499_g1
PLSCR1	Hs00275514_m1
TXNRD1	Hs00182418_m1
RAD50	Hs00194871_m1
INPPL1	Hs00155533_m1
PBX2	Hs00855025_s1
SSBP1	Hs00268409_m1
HSPCB	Hs00607336_gH
PDGFRA	Hs00183486_m1
ACOT4	Hs00745041_s1
DDOST	Hs00193263_m1
IGHA1	Hs00733892_m1
S100P	Hs00195584_m1
FAT	Hs00170627_m1
FGF2	Hs00960934_m1
INSM1	Hs00357871_s1
IRF5	Hs00158114_m1
SMARCD2	Hs00161987_m1
MAP2K2	Hs00360961_m1
HMBS	Hs00609297_m1
GAPDH	Hs99999905_m1
18S	Hs99999901_s1
IPO8	Hs00183533_m1

SEER description ICD-O-3

A cohort of patients diagnosed with primary breast cancer was retrieved from the Surveillance Epidemiology and End Results (SEER) database. The SEER database is an aggregate of registry data from specific geographic areas covering approximately 26 percent of the U.S. population and contains clinical, demographic, treatment, and follow-up information for a variety of cancers. The requirements for inclusion in this breast cancer study included a diagnosis of papillary carcinoma NOS (ICD-O-3 8050/3), spindle cell sarcoma (ICD-O-3 8801/3), carcinoma NOS (ICD-O-3 8010/3), infiltrating duct mixed with other types of ca. (ICD-O-3 8523/3), mucinous adenocarcinoma (ICD-O-3 8480/3), infiltrating duct carcinoma NOS (ICD-O-3 8500/3), or infiltrating lobular mixed with other types of ca. / infiltrating duct and lobular carcinoma / lobular carcinoma NOS (ICD-O-3 8524/3, 8522/3 and 8520/3) between the years 1988 and 2007, as well as available data on AJCC T-N-M (or tumor extend, nodes, size) information, tumor grade, survival, and treatment. A total of 400,978 patients met the inclusion criteria.

Table S2. Clinical characteristics of breast cancer patients in our patient cohorts and SEER database.

	**Our Study** (*n* = 60)	**SEER Database** (*n* = 400,978)
**Patient Age:**		
Median	60	61
Range	36-86	17-106
**Pathologic Characteristics:**		
Tumor Stage		
1	10 (16.67%)	214,474 (53.49%)
2	31 (51.67%)	157,177 (39.20%)
3	12 (20%)	23,942 (5.97%)
4	4 (6.67%)	1,304 (0.33%)
Missing info	3 (5%)	4,081 (1.02%)
Tumor Grade		
1	5 (8.33%)	67,661 (16.87%)
2	23 (36.67%)	183,857 (45.85%)
3	33 (55%)	149,460 (37.27%)
Missing info	0	0
**Histology**:		
Papillary carcinoma, NOS	1 (1.67%)	672 (0.17%)
Carcinoma, NOS	1 (1.67%)	1521 (0.38%)
Infiltrating duct mixed with other types of ca.	2 (3.33%)	7,771 (1.94%)
Mucinous adenocarcinoma	3 (5%)	8,766 (2.19%)
Infiltrating lobular mixed with other types of ca. / Infiltrating duct and lobular carcinoma / Lobular carcinoma, NOS	6 (10%)	58,448 (14.58%)
Infiltrating duct carcinoma, NOS	47 (78.33%)	323,800 (80.75%)

Table S3. Genes whose ΔC_T has a significant correlation ($p < 0.05$) with SEER patient survival. Correlations were obtained from SEER patients within three treatment groups (both surgery and radiation, surgery only and radiation only)

**Both surgery and radiation group (*n* = 195,717):**						
Gene	20-year survival		10-year survival		5-year survival	
	correlation	*p*-value	correlation	*p*-value	correlation	*p*-value
MCM2	**0.539**	0.017	**0.573**	0.010	**0.618**	0.005
RAD52	**0.692**	0.001	**0.676**	0.001	**0.600**	0.007
S100P	0.415	0.077	**0.477**	0.039	**0.501**	0.029
SMARCD2	**0.584**	0.009	**0.651**	0.003	**0.670**	0.002
PSMC3IP	0.427344	0.127	0.491	0.074	**0.542**	0.045
IGHA1	**0.528**	0.020	0.369	0.120	0.337	0.159
**Surgery only group (*n* = 204,434):**						
Gene	20-year survival		10-year survival		5-year survival	
	correlation	*p*-value	correlation	*p*-value	correlation	*p*-value
RAD52	**0.610**	0.006	**0.592**	0.008	**0.510**	0.026
SMARCD2	**0.486**	0.035	**0.581**	0.009	**0.574**	0.010
**Radiation only group (n = 827):**						
Gene	20-year survival		10-year survival		5-year survival	
	correlation	*p*-value	correlation	*p*-value	correlation	*p*-value
MCM2	**0.501**	0.029	**0.500**	0.029	**0.557**	0.013
RAD52	0.371	0.118	0.427	0.068	**0.480**	0.037
S100P	**0.533**	0.019	**0.545**	0.016	**0.615**	0.005
SMARCD2	0.423	0.071	0.448	0.054	**0.544**	0.016
PLSCR1	0.284	0.238	0.312	0.193	**0.510**	0.026
TOMM70A	0.308	0.199	0.283	0.241	**0.476**	0.039
TXNRD1	0.390	0.099	0.379	0.109	**0.531**	0.019

Table S4. Hazard ratio and 95% confidence interval of RAD52 and PBX2 in both overall survival univariate Cox model and recurrence-free univariate Cox model.

**Hazard Ratio in Overall Survival Model:**			
Protein	*IHC Score*	*Hazard Ratio*	*95% Confidence Interval*
RAD52	1	9.571	[1.321, 69.33]
	2	91.652	[1.745, 4806.652]
**Hazard Ratio in Recurrence-Free Survival Model:**			
Protein	*IHC Score*	*Hazard Ratio*	*95% Confidence Interval*
RAD52	1	3.471	[1.390, 8.668]
	2	12.049	[1.932, 75.134]
PBX2	1	1.704	[1.056, 2.750]
	2	2.903	[1.115, 7.563]
	3	4.946	[1.178, 20.797]
	4	8.428	[1.244, 57.191]

Table S5. SMARCD2 overall survival and recurrence-free survival IHC protein level COX results. *Warning message after Cox model: In fitter(X, Y, strats, offset, init, control, weights = weights, : Loglik converged before variable 1 ; beta may be infinite.

	OS coef	OS *p*-value	RFS coef	RFS *p*-value
SMARCD2	*19.7852351511077	*1	0.112586	0.85

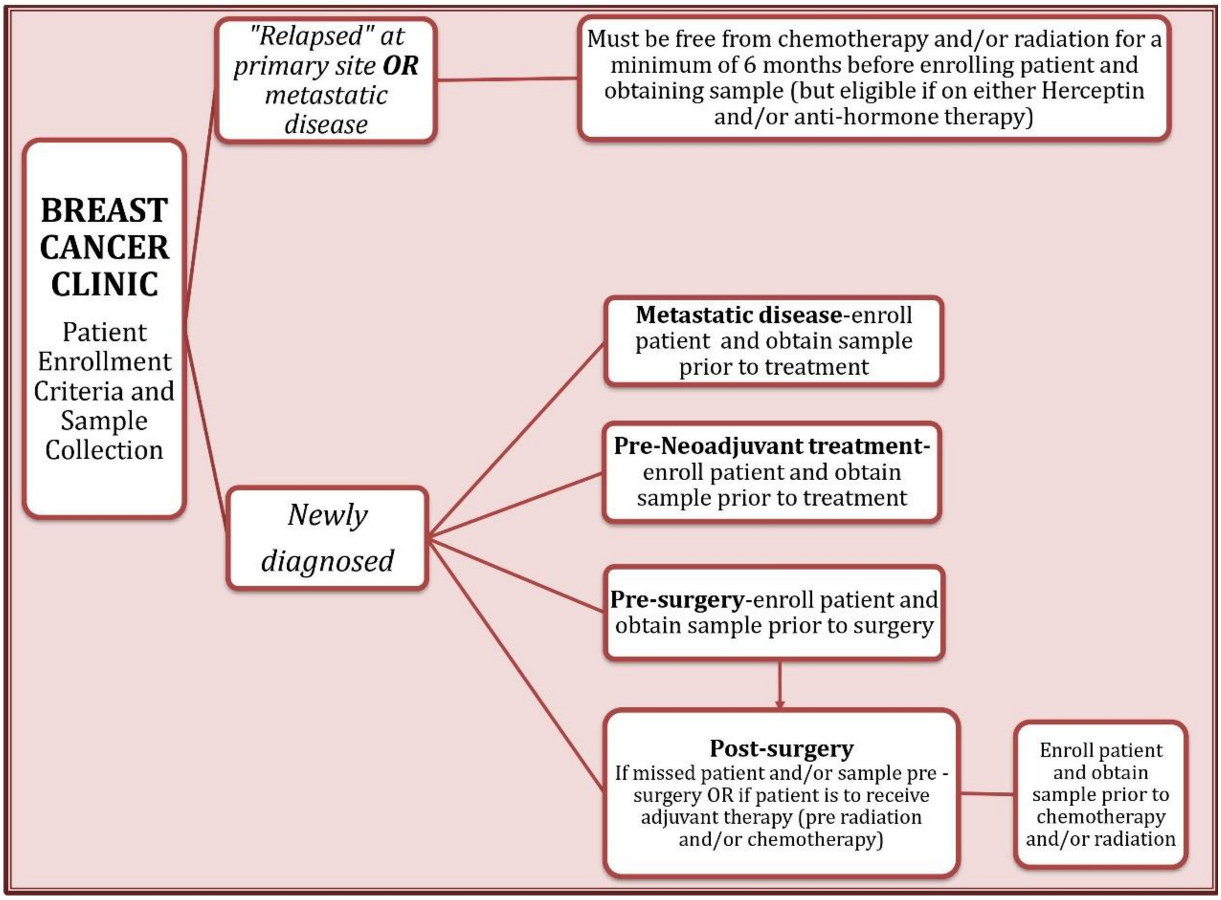

Figure S1. Breast cancer patient enrollment criteria.

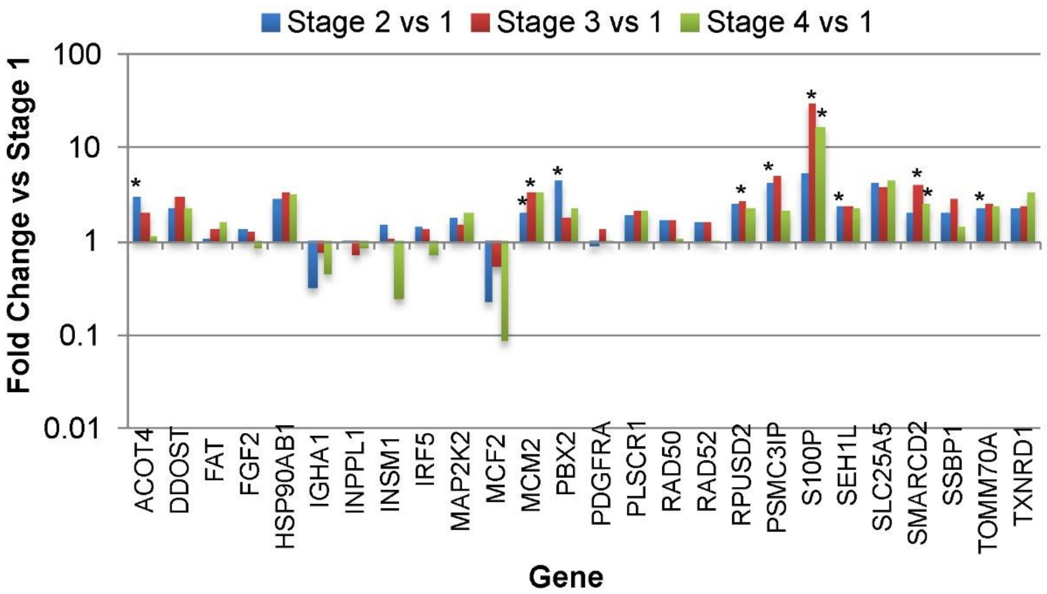

Figure S2. Stage fold change calculated with *HMBS* plate-wise. The average expression of *HMBS* in each plate was used as a house keeping gene measurement in the analysis. * $p < 0.05$ in two-sample t-tests.

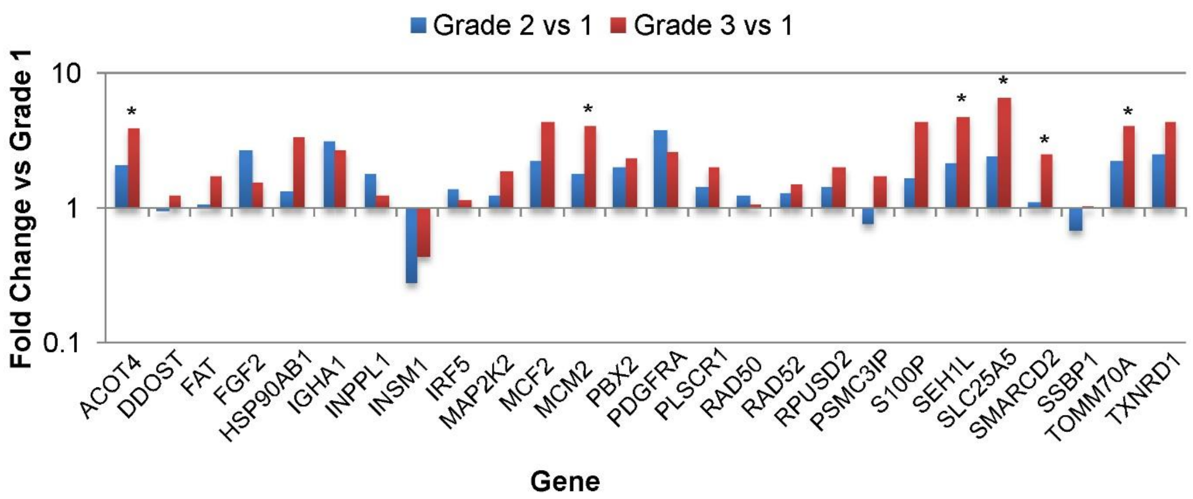

Figure S3. Grade fold change calculated with *HMBS* plate-wise. The average expression of *HMBS* in each plate was used as a house keeping gene measurement in the analysis. * $p < 0.05$ in two-sample *t*-tests.

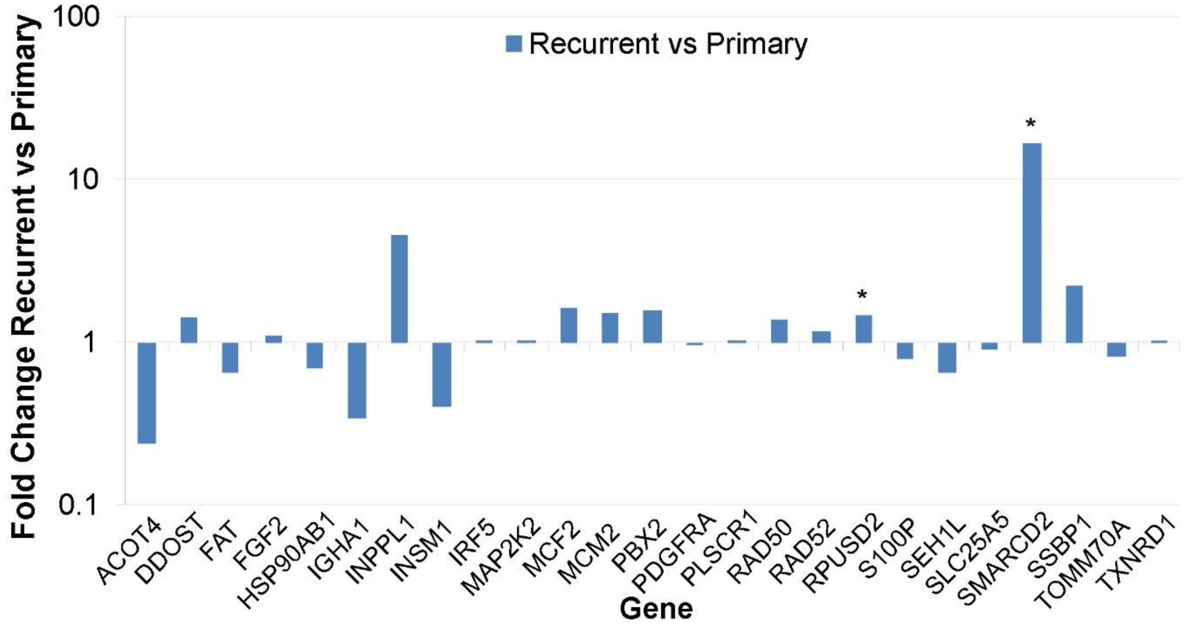

Figure S4. Fold change recurrent vs primary breast cancer for grade 3 samples (25 genes) calculated with *HMBS* sample-wise normalization. The average expression of *HMBS* in each sample was used as a house keeping gene measurement in the analysis. PSMC3IP was not included, since it was only measured in recurrent samples. Sample wise normalization was used since the recurrent samples were in different plates. There were 4 recurrence cancers with grade 3; 33 primary cancers with grade 3. * $p < 0.05$ in two-sample *t*-tests.

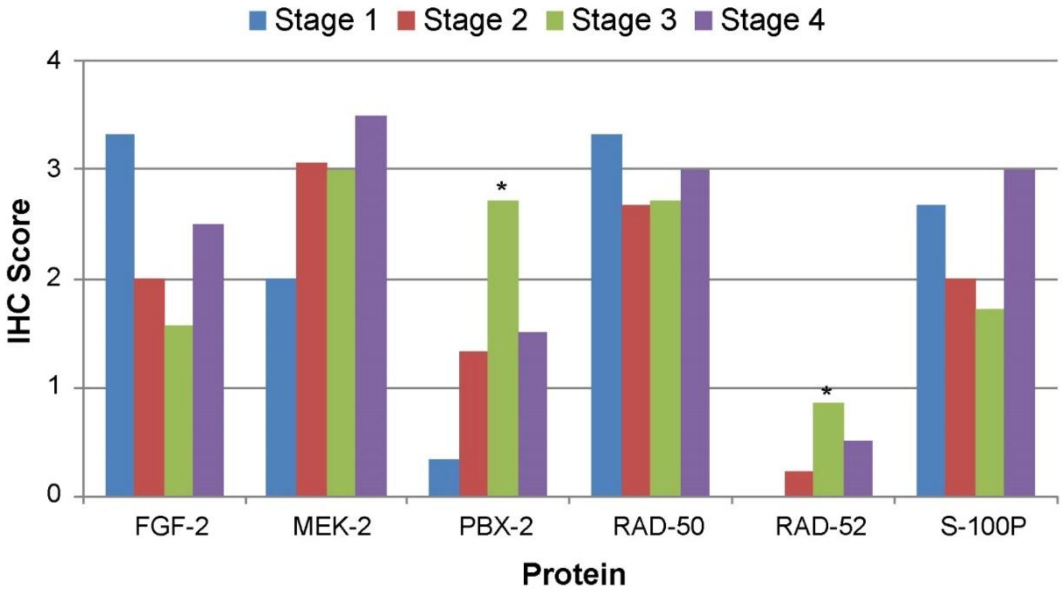

Figure S5. Average protein expression measured with immunohistochemistry (IHC) score in each breast cancer stage. *$p < 0.05$ in ANOVA tests.

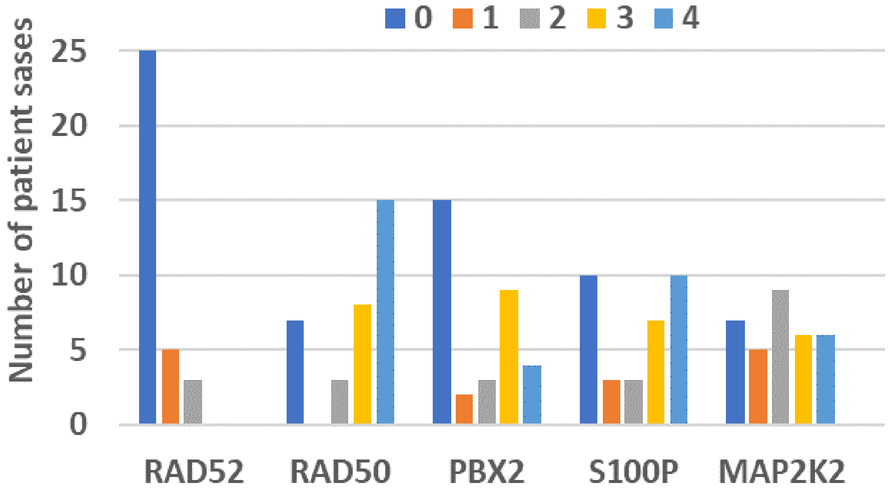

Figure S6. Protein expression levels of RAD52, RAD50, PBX2, S100P, and MAP2K2 scored in immunohistochemistry assays of invasive breast cancer tumor samples (n=33). 0 = no staining; 1 = equivocal staining; 2 = weak staining; 3 = moderate staining; 4 = strong staining.

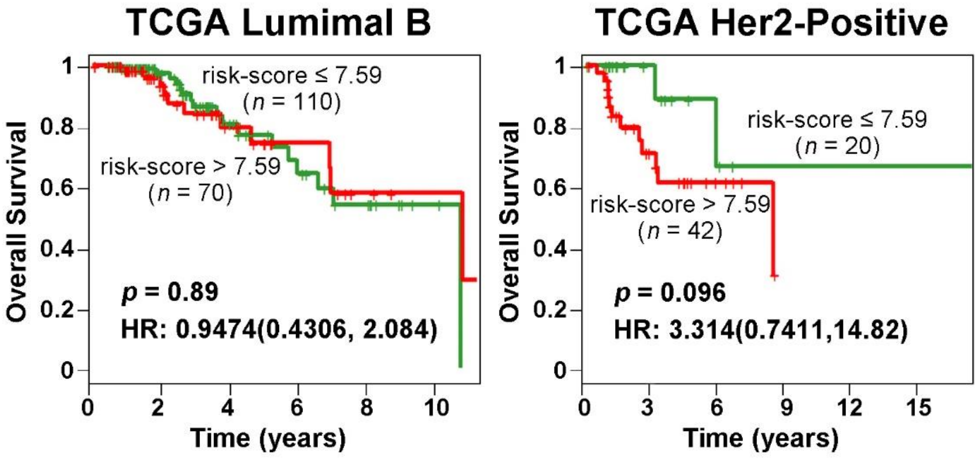

Figure S7. For the subtypes Luminal B and Her2-positive patients in TCGA-BRCA, the low-risk and high-risk patient group did not have significantly different survival. The plots showed the survival outcome of the first 10 years after surgery.

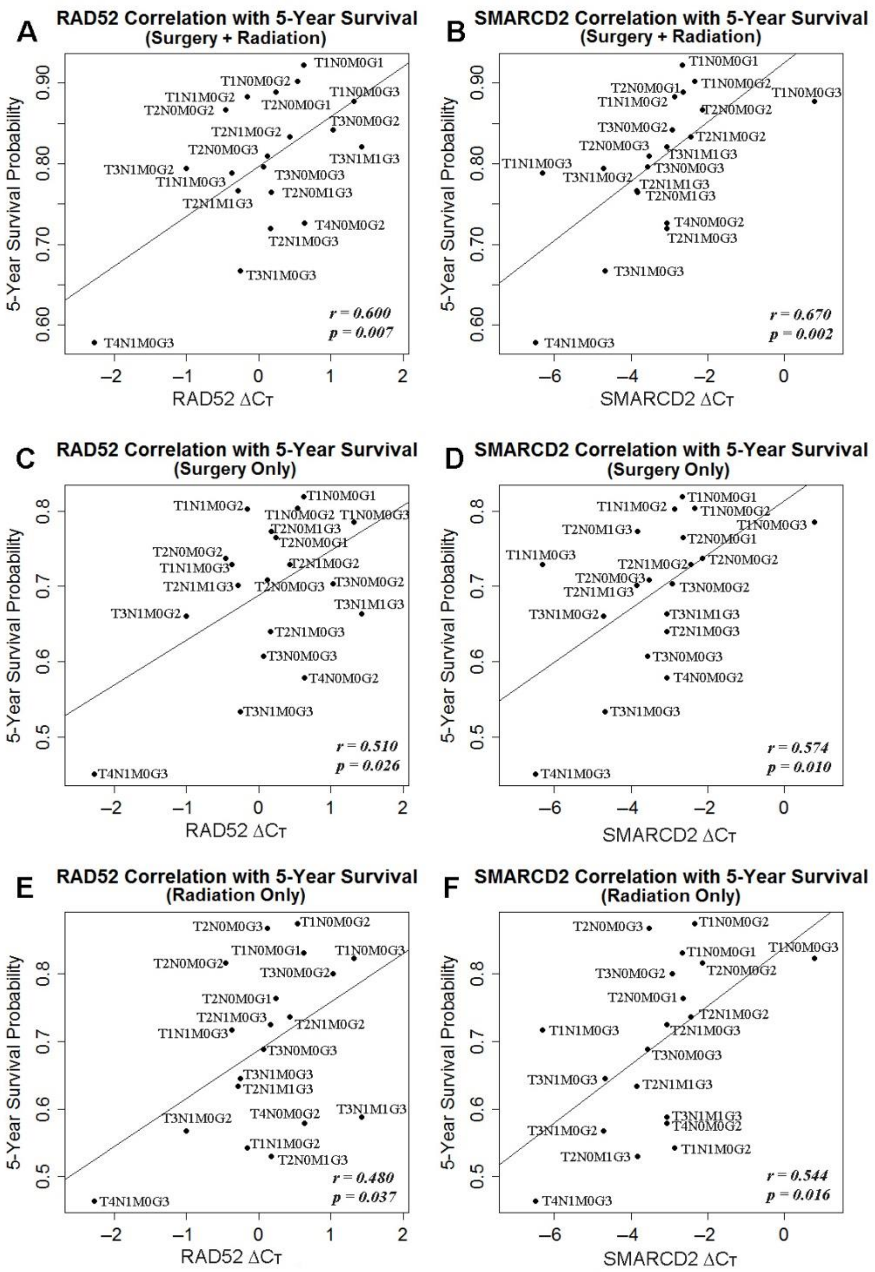

Figure S8. Correlations between gene (RAD52 and SMARCD2) ΔCT and 5-year survival in the three treatment groups (both surgery and radiation, surgery only and radiation only). The T-N-M-grade category is labeled beside each data point.
